# Insight into Olfactory Learning, Memory, and Mortality of *Apis mellifera jemenitica* after Exposure to Acetamiprid Insecticide

**DOI:** 10.3390/insects15070473

**Published:** 2024-06-25

**Authors:** Mohamedazim I. B. Abuagla, Javaid Iqbal, Hael S. A. Raweh, Abdulaziz S. Alqarni

**Affiliations:** Department of Plant Protection, College of Food and Agriculture Sciences, King Saud University, P.O. Box 2460, Riyadh 11451, Saudi Arabia; mabuagla@ksu.edu.sa (M.I.B.A.); jiqbal@ksu.edu.sa (J.I.);

**Keywords:** honey bee, neonicotinoid, insecticide, lethal toxicity, oral routes, topical routes, mortality, cognitive skills

## Abstract

**Simple Summary:**

This study highlights the significant impact of acetamiprid, a neonicotinoid insecticide, on the survival and formation of olfactory appetitive learning and memory in *Apis mellifera jemenitica*. The insecticide exhibits deleterious effects on honey bees through both topical and oral exposure routes. This information holds significant value for devising comprehensive strategies aimed at better enhancing honey bee foraging activities within agricultural landscapes treated with chemicals. By understanding the specific impacts of insecticides against honey bees, we can develop targeted approaches to mitigate adverse effects, preserve honey bee populations, and sustain pollination services that are crucial for ecosystem health and agricultural productivity.

**Abstract:**

The honey bee, a significant crop pollinator, encounters pesticides through various routes of exposure during foraging and flower visitation. Considering the potential threat of pesticide poisoning, the indigenous Saudi bee *Apis mellifera jemenitica* is susceptible to the risks associated with acetamiprid, a neonicotinoid insecticide. This study investigates the acetamiprid-induced effects on the survival, olfactory learning, and memory formation of *A. m. jemenitica* through two exposure routes: topical application and oral ingestion. Field-realistic and serially diluted concentrations (100, 50, 25, and 10 ppm) of acetamiprid led to notable mortality at 4, 12, 24, and 48 h after treatment, with peak mortality observed at 24 h and 48 h for both exposure routes. Bee mortality was concentration-dependent, increasing with the rising concentration of acetamiprid at the tested time intervals. Food consumption following oral exposure exhibited a concentration-dependent pattern, steadily decreasing with increasing concentrations of acetamiprid. Oral exposure resulted in a substantially higher cumulative mortality (55%) compared to topical exposure (15%), indicating a significant disparity in bee mortality between the two exposure routes. The 24 h post-treatment LC_50_ values for acetamiprid were 160.33 and 12.76 ppm for topical application and oral ingestion, respectively. The sublethal concentrations (LC_10_, LC_20_, and LC_30_) of acetamiprid were 15.23, 34.18, and 61.20 ppm, respectively, following topical exposure, and 2.85, 4.77, and 6.91 ppm, respectively, following oral exposure. The sublethal concentrations of acetamiprid significantly decreased learning during the 2nd–3rd conditioning trials and impaired memory formation at 2, 12, and 24 h following both topical and oral exposure routes, compared to the control bees. Notably, the sublethal concentrations were equally effective in impairing bee learning and memory. Taken together, acetamiprid exposure adversely affected bee survival, hindered learning, and impaired the memory retention of learned tasks.

## 1. Introduction

Honey bees are significant crop pollinators that produce wax, honey, propolis, royal jelly, pollen, beebread, and bee venom [1,2,3,4]. Bees also play a vital role in preserving the ecological equilibrium [5]. In recent decades, a noticeable decline has been observed in honey bee populations [6]. Threats to bees include modern agriculture intensification, habitat degradation and fragmentation due to deforestation, human modification in land use, inadequate nutrition for pollinators, climatic change, and global warming [7,8]. In addition, diseases (bacteria, viruses, fungus, and protozoa), *Varroa destructor* parasitic mite infestation, beekeeping management practices, and agrochemical use can all have adverse impacts on honey bee populations [9,10,11,12]. Pesticides can be harmful to pollinators including bees, butterflies, bumblebees, and other non-target natural enemies [13,14].

Pesticide usage in agriculture also poses deleterious effects on honey bee development, reproduction, and behavioral activities [15,16]. Agrochemicals are highly toxic and have rapid knockdown effects against insects, including pollinators at lethal or sublethal concentrations [17]. Lethal effects due to acute exposure to insecticides cause immediate honey bee mortality [18]. Sublethal concentrations can cause impairments to navigation and foraging abilities, communication, reduced learning and memory, and altered reproductive success in honey bees [19,20,21]. 

Neonicotinoid insecticides are widely used in agriculture due to their high killing efficiency against a wide range of insect pests [22,23,24]. Neonicotinoids are systemic pesticides used in different ways such as seed treatments, soil drenches, foliar sprays, and coatings on granular products [25]. They function through the central nervous system of insects through interaction with nicotinic acetylcholine receptors, disrupting normal nerve signaling and leading to paralysis in the targeted pests [26,27]. Neonicotinoid insecticides are extensively used across the globe to control sucking-type insects that attack various crops, vegetables, fruits, cotton, grapes, ornamental plants, mosquitos, and locusts [28,29]. These insecticides have shown devastating influence on certain honey bee behaviors, including foraging, pollination, longevity, queen reproductive success, learning capacity, and colony fitness [30,31,32]. Acetamiprid is a broad-spectrum neonicotinoid frequently used against agricultural pests and acts on nicotinic cholinergic receptors in insects [5,33]. Acetamiprid also has negative impacts on non-target organisms including honey bees [34]. It exhibits high toxicity to honey bees and can affect the central nervous system of bees, resulting in paralysis and death [35,36]. 

In Saudi Arabia, synthetic pesticides are commonly used in agriculture and in mosquito control programs [37]. Acetamiprid is used against flies, ants, and cockroaches, red palm weevils, whiteflies, aphids, leaf- and plant hoppers, thrips, and mosquito larvae worldwide, including in Saudi Arabia [38,39]. The Saudi Food and Drug Authority has officially registered acetamiprid to control certain insects of public interest in Saudi Arabia [40]. High percentages of pesticide residues above the recommended limit were found in agricultural products in the Al-Qassim area of Saudi Arabia [41]. Furthermore, acetamiprid residues were detected in forty vegetable samples from the city of Unaizah, Saudi Arabia [42]. 

Beekeeping (apiculture) has a long history in Saudi Arabia and plays a significant role in the country’s agricultural sector [43]. It contributes to the local economy, provides employment opportunities, and supports agricultural sustainability [44]. *Apis mellifera jemenitica* Ruttner, 1976, is the dominant indigenous bee for honey production and pollination services [45]. *A. m. jemenitica* originates from the Arabian Peninsula and tropical Africa and is characterized by high thermal tolerance, foraging in extreme conditions due to a relatively small body, and survival in drought with little food storage [45]. 

Honey bees possess a sophisticated olfactory system, and their antennae are important for the process of smell [46]. The sense of smell plays a vital role for honey bee foragers, allowing them to communicate, navigate, locate food sources, mate, and engage in social interactions within their colony [47,48]. Olfactory functioning is an essential component of bee survival and contributes significantly to the health, fitness, and productivity of a bee colony [49,50].

Therefore, any changes in olfaction induced by insecticidal exposure can affect waggle dance, orientation, reproduction, and the forager performance (foraging for nectar, pollen, and water collection) [51,52,53]. It is imperative to consider an insecticide’s concentration, chemical nature, exposure duration, and type when analyzing its impact on honey bees [54,55,56]. Bees encounter agrochemicals through multiple routes, such as topical exposure (directly sprayed crops) and oral intake through contaminated pollen, nectar, and water sources [57,58]. Systemic insecticides that are taken up by plants can be harmful to bees through their intake of plant tissues, including pollen and nectar [59]. Insecticides can also drift into hives from nearby agricultural areas [18,60]. 

We hypothesized that acetamiprid, a neonicotinoid insecticide, may have detrimental effects on honey bees’ survival and olfactory cognitive abilities. Therefore, this study aimed to investigate the toxicity of field-realistic and lethal acetamiprid concentrations against a Saudi-native honey bee, *A. m. jemenitica,* through two exposure methods (topical and oral) at different time periods. Furthermore, we investigated the deleterious effects of sublethal acetamiprid concentrations on olfactory learning and memory formation in *A. m. jemenitica* under controlled conditions. 

This study provides valuable insights by comprehensively assessing the impact of the acetamiprid insecticide on olfactory learning in a Saudi-native honey bee, *A. m. jemenitica*, thus highlighting the harmful effects of neonicotinoid insecticides on bees through multiple exposure routes. The integration of toxicity testing with behavioral assays presents a novel approach to studying pesticide effects on honey bee cognition, providing insights into the ecological consequences of pesticide use in agricultural landscapes and its implications for surrounding beekeeping environments.

## 2. Materials and Methods

Colonies of *Apis mellifera jemenitica*, a honey bee native to Saudi Arabia, were maintained at an educational farm located at the coordinates 24°44′14.2″ N 46°37′09.9″ E within the King Saud University, Riyadh, Saudi Arabia. The hives were kept free from pathogen infestations and insecticidal applications. Bees returning from foraging were randomly caught at the entrance to the hive [61,62]. Using a fine brush, the bees were placed in wooden cages [63], and brought to the laboratory for subsequent analyses. The bees were directly kept in an incubator at 25 ± 2 °C and 60 ± 10% RH for 2 h [63,64,65].

### 2.1. Insecticide Application, Bee Mortality, and Lethal Concentrations

Commercially available acetamiprid (Cetam^®^ 20SL) was procured from a market in Riyadh, Saudi Arabia. A field-realistic concentration of acetamiprid (100 ppm), was prepared as recommended by the manufacturer, along with three sequentially diluted concentrations (50, 25, and 10 ppm) sourced from the pesticide bottle. The chosen formulation of acetamiprid was prescribed by the manufacturer (Al-Burj Agrivet Pesticide Manufacturing Co., Ltd., Amman, Jordan) and distributor (Saudi United Fertilizer Co., Jeddah, Saudi Arabia) to use on vegetables and fruits against insect pests such as aphids, leafminers, whiteflies, mealybugs, leafhoppers, and various other insects in the field. 

Bee mortality was evaluated using two distinct exposure routes for the insecticide: topical application and oral feeding. The serial dilutions were formulated in acetone (for topical application) and mixed with 50% *w*/*v* sucrose (for oral ingestion). The control group of bees was exposed to acetone alone and a 50% *w*/*v* sucrose solution free of insecticide by topical and oral exposure, respectivley. The lethal concentration (LC_50_) was calculated from the mortality of bees observed at different times (12, 24, and 48 h) in response to the tested dilutions. 

#### 2.1.1. Topical Application

For topical exposure, 1 µL of each acetamiprid serial dilution (100, 50, 25, and 10 ppm) formulated with acetone was applied dorsally on the thorax of the bees, following the methodologies outlined in previous studies [63,66]. Acetone alone served as the control treatment in the control group of bees [62]. Ten bees were treated for each tested concentration and placed into individual plastic containers [67], each equipped with two plastic syringes of 5 mL. One syringe was designated for the sucrose solution and the other for water, serving as feeder units [63,68]. Fresh water and sucrose were both replenished daily throughout the experiment. The treated bees and their respective plastic containers were placed in an incubator set at 25 ± 2 °C and 60 ± 10% RH. The mortality of the bees exposed to various acetamiprid dilutions was recorded by individually recording the number of dead bees from all containers at various time points after treatment (4, 12, 24, and 48 h). Four replicates were carried out for each serial dilution of acetamiprid. 

#### 2.1.2. Oral Ingestion

To investigate oral exposure, an acute oral toxicity test was conducted on the honey bees, following standard protocols [64,69]. The bees were kept in wooden cages and deprived of food to induce a substantial level of starvation, which facilitated efficient feeding within a short timeframe [70]. Ten bees were treated for each tested concentration and placed into individual plastic containers [67]. Serial dilutions (100, 50, 25, and 10 ppm) of acetamiprid were formulated using 50% *w*/*v* sucrose solution. The bees were divided into five groups in separate plastic containers. Each group of bees was administrated 200 µL of a sucrose solution containing a dilution of acetamiprid [69,71]. The bees were allowed to feed for 4 h and were kept in an incubator set at 25 ± 2 °C and 60 ± 10% RH [69,72]. The control bees received a 50% sucrose solution without insecticide [69]. After 4 h, the insecticide-contaminated sucrose solution was removed from each plastic container. The bees were then left without food for 1 h to digest the provided food [65]. The quantity of acetamiprid-contaminated food consumed by each group of bees at different dilutions after 4 h of feeding was noted, and the percentage food consumption was then calculated. Subsequently, the bees were provided with 50% sucrose solution (*w*/*v*) and water ad libitum [69] in two designated syringes for the sucrose solution and water, serving as feeder units [63]. Both water and sucrose were freshly replenished daily throughout the experiment. The number of dead bees were individually recorded from all containers at different durations after treatment (4, 12, 24, and 48 h). The mortality of the bees exposed to various dilutions of acetamiprid was calculated, and each experiment with serial dilutions of acetamiprid was replicated four times [72]. The percentage mortality of the honey bees, corrected for the control treatment mortality, was calculated for each tested dilution following both topical and oral exposure routes to the insecticide at various time intervals. The lethal (LC_50_ and LC_90_) and sublethal (LC_10_, LC_20_, and LC_30_) concentrations of acetamiprid were determined at 12, 24, and 48 h. The term sublethal, as observed in the scientific literature, describes concentrations that are lethal to a small portion of the population but generally considered sublethal for the majority of the population [73].

### 2.2. Exploring Olfactory Learning and Memory

The incoming forager bees were randomly captured at the hive entrance [63,74]. The bees were immobilized on ice for 3–4 min, and then harnessed in plastic tubes, using producers outlined in previous studies [61,75]. The bee heads were fixed with dental wax to restrict head movement. Following 30 min of fixation, the harnessed bees were fed with 0.5 M sucrose and kept overnight in a dark, moist environment maintained at 25 ± 2.0 °C and 50 ± 10% RH to ensure their survival. Ten minutes before the insecticide application and learning trials, the bees were initially motivated by touching a 0.5 M sucrose solution to their antennae without providing food. The bees that exhibited a response by extending their proboscis to the initial stimulus were included in the subsequent learning experiments [62,75]. Individuals that did not display any response to the initial sugar stimulus were omitted [75,76]. Afterward, the harnessed adult forager bees were subjected to sublethal concentrations of acetamiprid, established at 24 h (LC_10_, LC_20_, and LC_30_), administrated through topical and oral routes. Three sublethal concentrations, each of 1 µL, were administered topically or orally one hour before the learning trials [62]. The control group of bees was administered acetone alone for topical exposure and a 50% sucrose solution for oral exposure. The treated bees experienced three consecutive learning trials at 10 min intervals adhering to the standard olfactory associative learning protocol for exploring learning and memory formation. In this protocol, odor stimulus (clove oil) served as the conditioning stimulus (CS), which was paired with an unconditioned stimulus (UCS) represented by an appetitive reward of 1 M sucrose [61,75]. Memory formation was evaluated at 2, 12, and 24 h following the learning process, utilizing only CS (clove oil) [77]. The proboscis extension response (PER) served as an indicator for assessing the learning and memory capabilities of the honey bees [78]. A positive PER was noted when the bees fully extended their proboscis in reaction to the odor stimulus, while a lack of response to the odor was recorded as negative [75,79]. The harnessed bees were then continuously fed with 0.5 M sucrose every 4 h to ensure their survival during the experiment period [61,62]. The PER (%) in both the learning trials and memory testing were calculated to quantify the extent of learning and memory formation. Each dilution experiment with a sublethal acetamiprid concentration was replicated four times, with approximately twenty bees included in each replication.

### 2.3. Statistical Analysis

The mortality data on all tested acetamiprid dilutions were utilized to determine both lethal and sublethal concentrations. The corrected mortality was computed with Abbott’s formula [63,80]. Lethal concentrations were determined with a 95% confidence interval for upper and lower limits through Probit analysis [81], aided using LdP Line software [82]. An analysis of variance (ANOVA) was conducted using SAS software (version 9.4) to analyze the data on bee mortality and food consumption. Subsequently, means were compared with the Least Significant Difference (LSD) test. The assumptions required for the parametric analyses were verified. The data on PER during honey bee learning and memory formation were analyzed using Fisher’s exact test or the Chi-square (χ^2^) test, considering significance at *p* < 0.05.

## 3. Results

Acetamiprid showed toxicity against *A*. *m. jemenitica* in terms of percentage mortality of bees under laboratory conditions. Sublethal concentrations of acetamiprid were toxic, affecting the olfactory learning and memory formation of *A*. *m. jemenitica* when applied through topical and oral routes. 

### 3.1. Topical Exposure to Acetamiprid

**Figure 1:**
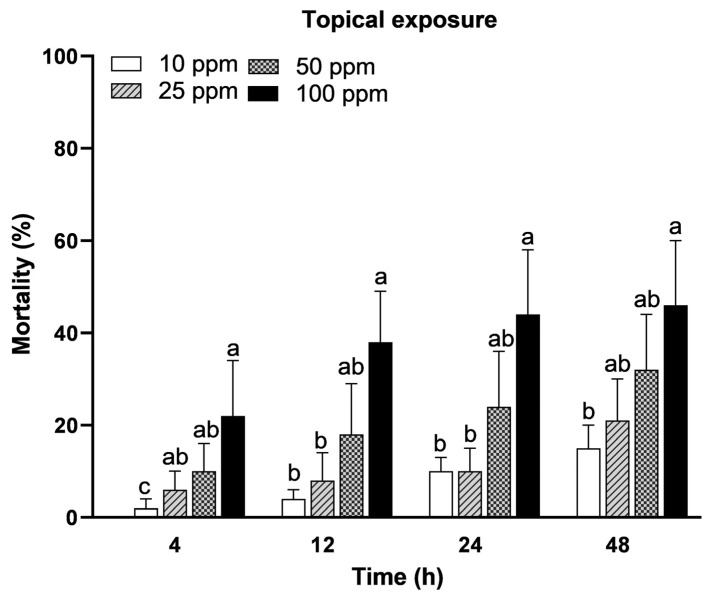
Mortality of *Apis mellifera jemenitica* in response to topical exposure to acetamiprid. Different letters represent significant difference (*p* < 0.05) at each tested time period. The bars represent the standard error of mean.

The topical application of the tested concentrations of acetamiprid (10, 25, 50, and 100 ppm) revealed significant differences (F = 20.45; *p* < 0.0001) in *A. m. jemenitica* percent mortality. An increase in the acetamiprid concentration resulted in increased mortality at all the tested times frames (4, 12, 24, and 48 h). The highest concentration (100 ppm) resulted in significant mortality percentages of 22%, 38%, 44%, and 46% at 4, 12, 24, and 48 h, respectively. This was followed by the 50 ppm concentration, which recorded mortalities of 10%, 18%, 24%, and 32% at the same time intervals. The 25 ppm acetamiprid concentration-induced mortalities were 6, 8, 10, and 21% at 4, 12, 24, and 48 h, respectively (Figure 1). The tested concentrations showed significantly different results from the control group. The control group had zero values, so these are not given in the graph. All the tested concentrations were significantly different at 12 h (F = 4.15; *p* = 0.0131), 24 h (F = 3.81; *p* = 0.0185), and 48 h (F = 3.59; *p* = 0.0231) after treatment. 

### 3.2. Oral Feeding

**Figure 2:**
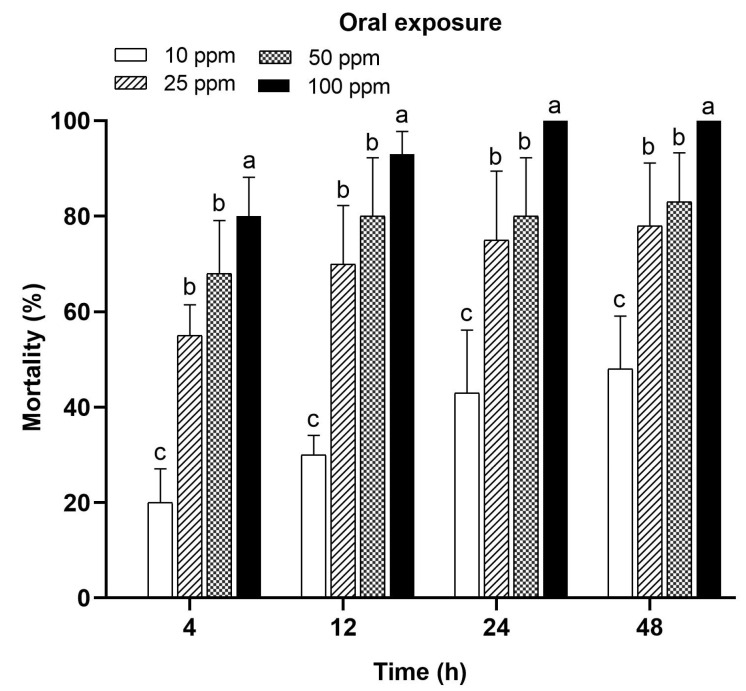
Mortality of *Apis mellifera jemenitica* in response to oral feeding of acetamiprid. Different letters represent the significant difference (*p* < 0.05) at each tested time period. The bars represent the standard error of mean.

Oral exposures to acetamiprid revealed high *A. m. jemenitica* mortality at all the tested concentrations (10, 25, 50, and 100 ppm) during the different time periods (4, 12, 24, and 48 h). Like topical exposure, the increasing acetamiprid concentrations resulted in a gradual increase in mortality. The highest concentration (100 ppm) caused high mortality (80, 93, 100, and 100%), followed by 50 ppm (68, 80, 80, and 83%) and 25 ppm (55, 70, 75, and 78%) at 4, 12, 24, and 48 h, respectively. The lowest mortality percent (20, 30, 43, and 48%) was recorded with 10 ppm at 4, 12, 24, and 48 h, respectively (Figure 2). The tested concentrations showed significantly different results from the control group. The control group had zero values, so these are not given in the graph. All the tested concentrations were significantly different at 12 h (F = 12.74; *p* = 0.001), 24 h (F = 14.42; *p* = 0.0001), and 48 h (F = 19.14; *p* = 0.0001) after treatment.

### 3.3. Food Consumption after Oral Exposure

**Figure 3:**
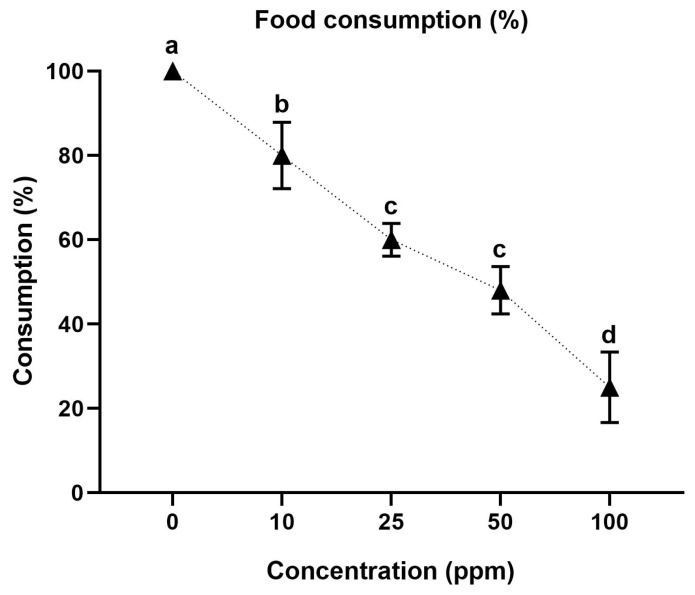
Effects of oral feeding of acetamiprid on percent food consumption of *Apis mellifera jemenitica*. Different statistical letters denote a significant difference (*p* < 0.05) among tested concentrations. The bars represent the standard error of the mean.

The oral feeding of the different tested acetamiprid concentrations (10, 25, 50, and 100 ppm) significantly reduced the food consumption of *A. m. jemenitica* compared to the control group. A significant difference (F = 22.1; *p* < 0.0001) among the tested concentrations for food consumption was recorded. Food consumption gradually reduced with an increase in the tested insecticide concentration. The highest food consumption (100%) was noted in the control bees fed with sucrose (without any insecticide). Food consumption gradually reduced (80, 60, and 48%) with the tested acetamiprid concentrations (10, 25, and 50 ppm, respectively). The lowest food consumption (26%) was observed with the highest tested concentration (100 ppm) (Figure 3). It is worth noting that the oral exposure to higher doses of acetamiprid resulted in the lowest food intake but was also lethal, causing mortality (Figure 2).

### 3.4. Lethal Concentrations of Acetamiprid

**Figure 4:**
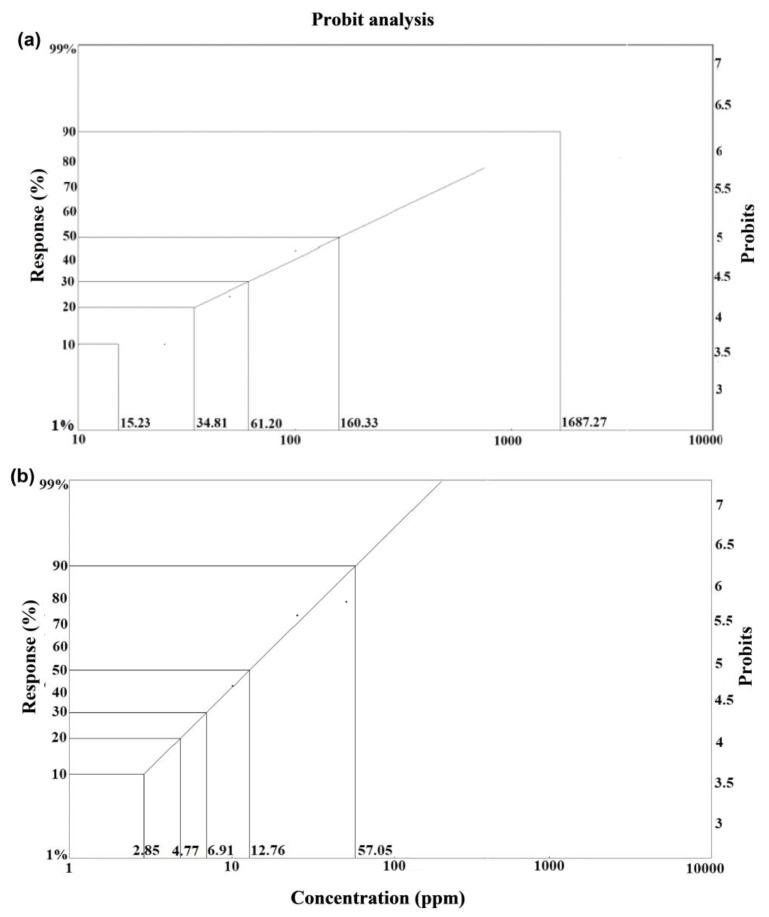
Probit analysis reveals lethal and sublethal concentrations of acetamiprid at 24 h after (**a**) topical exposure and (**b**) oral exposure.

The lethal concentration (LC_50_) values for topical and oral exposure were calculated at different time periods. The LC_50_ values were 172.67, 160.33, and 141.94 ppm for topical exposure and 16.70, 12.76, and 11.06 ppm for oral exposure at 12, 24, and 48 h, respectively (Table 1). Table 2 shows the sublethal concentrations (LC_10_, LC_20_, and LC_30_) determined at 12, 24, and 48 h after topical or oral exposure to acetamiprid. The graph in Figure 4 shows the lethal and sublethal acetamiprid concentrations at 24 h after topical (Figure 4a) and oral exposure (Figure 4b) against *A. m. jemenitica*.

### 3.5. Comparison between Mortality after Topical and Oral Exposure to Acetamiprid

**Figure 5:**
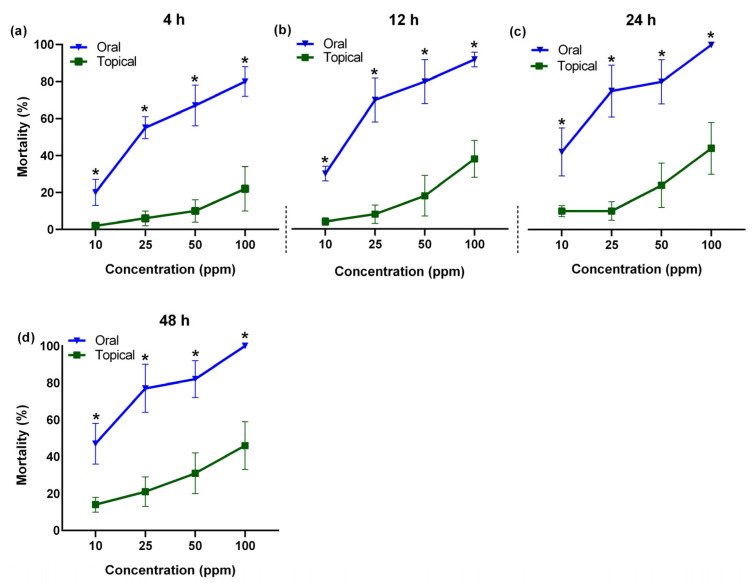
Comparison of acetamiprid concentrations via two exposure routes and mortality of *Apis mellifera jemenitica* at various time intervals: (**a**) 4 h, (**b**) 12 h, (**c**) 24 h, and (**d**) 48 h. Asterisks denote a significant difference between groups at each tested time point (*p* < 0.05, *t* test). The bars represent the standard error of the mean.

**Figure 6:**
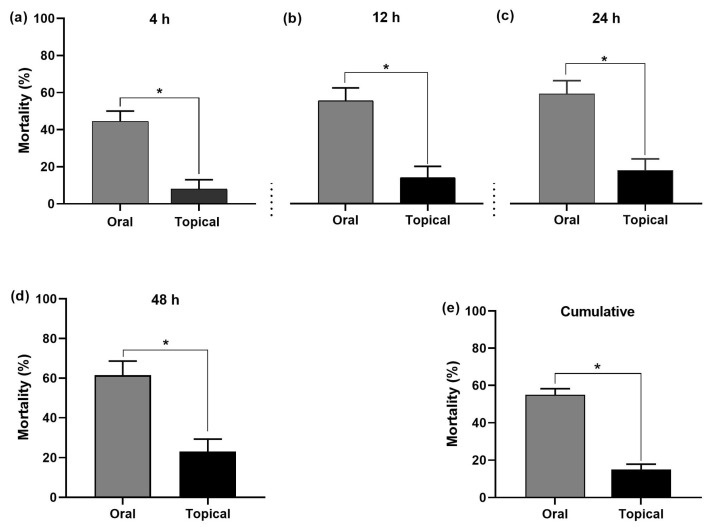
Comparison of acetamiprid exposure routes on the mortality of *Apis mellifera jemenitica* at (**a**) 4 h (**b**) 12 h, (**c**) 24 h, and (**d**) 48 h. (**e**) Cumulative mortality. Asterisks denote a significant difference between groups at each tested time point (*p* < 0.05, *t* test). The bars represent the standard error of the mean.

Comparison of all the diluted acetamiprid concentrations (10, 25, 50, and 100 ppm) revealed significantly higher bee mortality after oral exposure compared to topical exposure at the tested time points (Figure 5). The oral acetamiprid exposure showed a higher bee mortality (%) than the topical exposure at the tested time intervals after treatment (4, 12, 24, and 48 h) (Figure 6). The mortality percentages at 4, 12, 24, and 48 h were 44, 54, 60, and 62% after oral exposure, respectively, compared to 8, 14, 18 and 23% after topical exposure (Figure 6). *A. m. jemenitica* revealed a significantly high cumulative mortality after acetamiprid oral exposure (55%) in comparison to topical acetamiprid exposure (15%) (Figure 6e). 

### 3.6. Olfactory Behavioral Analysis (Learning and Memory Formation)

A classical proboscis extension response (PER) analysis was used to investigate the induced effects of sublethal acetamiprid concentrations on the learning and memory formation of *A. m. jemenitica* in response to topical and oral routes of insecticide exposure.

#### 3.6.1. Topical Exposure and Olfactory Behavior

**Figure 7:**
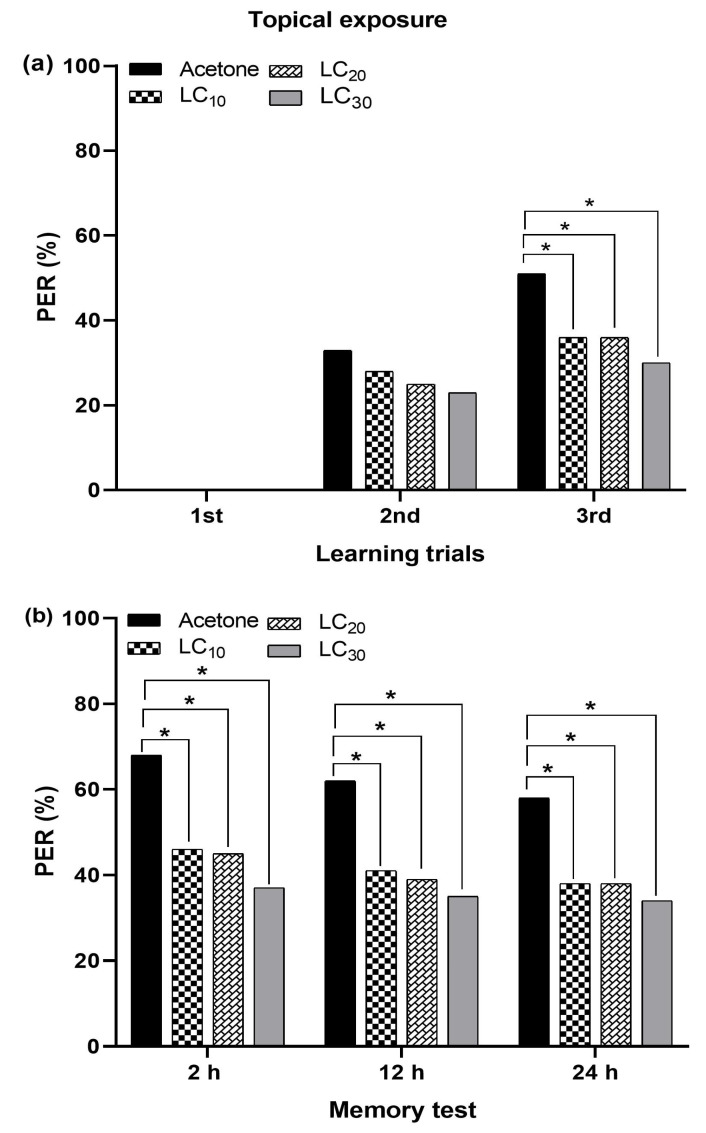
Topical administration of acetamiprid to *Apis mellifera jemenitica*. PER in (**a**) learning trials and (**b**) memory formation. Significant differences between control and insecticide-treated bees are denoted by asterisks (Fisher’s exact test/χ^2^ test; * *p* < 0.05).

The sublethal concentrations (LC_10_ = 15.23, LC_20_ = 34.18, and LC_30_ = 61.20 ppm) of acetamiprid negatively affected the olfactory learning and memory formation, expressed as the PER, when applied topically. No PER was observed during the first learning trial in both the control and treated bees. The change in learning was concentration-dependent, with a higher sublethal concentration (LC_30_) causing the highest reduction, followed by LC_20_ and LC_10_ as compared to the control group, which showed the highest level of learning. In the treated bees, a decrease in PER gradually occurred for all tested sublethal concentrations. After the second learning trial, the PER in treated bees revealed no significant differences among all the tested sublethal concentrations. During the third learning trial, a significant difference in the PER in treated bees was observed for all the tested sublethal concentrations of acetamiprid, compared to the control group of bees (Figure 7a). The PER in treated bees was significantly reduced at 2 h, 12 h, and 24 h after topical exposure to sublethal acetamiprid concentrations, compared to those of control bees during memory formation. The control group of bees showed higher PERs (68%, 62%, and 58%) at 2, 12, and 24 h, respectively, compared to treated bees exposed to the tested sublethal concentrations. All the tested sublethal concentrations caused an equal reduction in memory formation at 2, 12, and 24 h (Figure 7b). Thus, a lower sublethal concentration (LC_10_) can exert the same effects on memory formation as higher sublethal concentrations (LC_20_ and LC_30_).

#### 3.6.2. Oral Exposure and Olfactory Behavior

**Figure 8:**
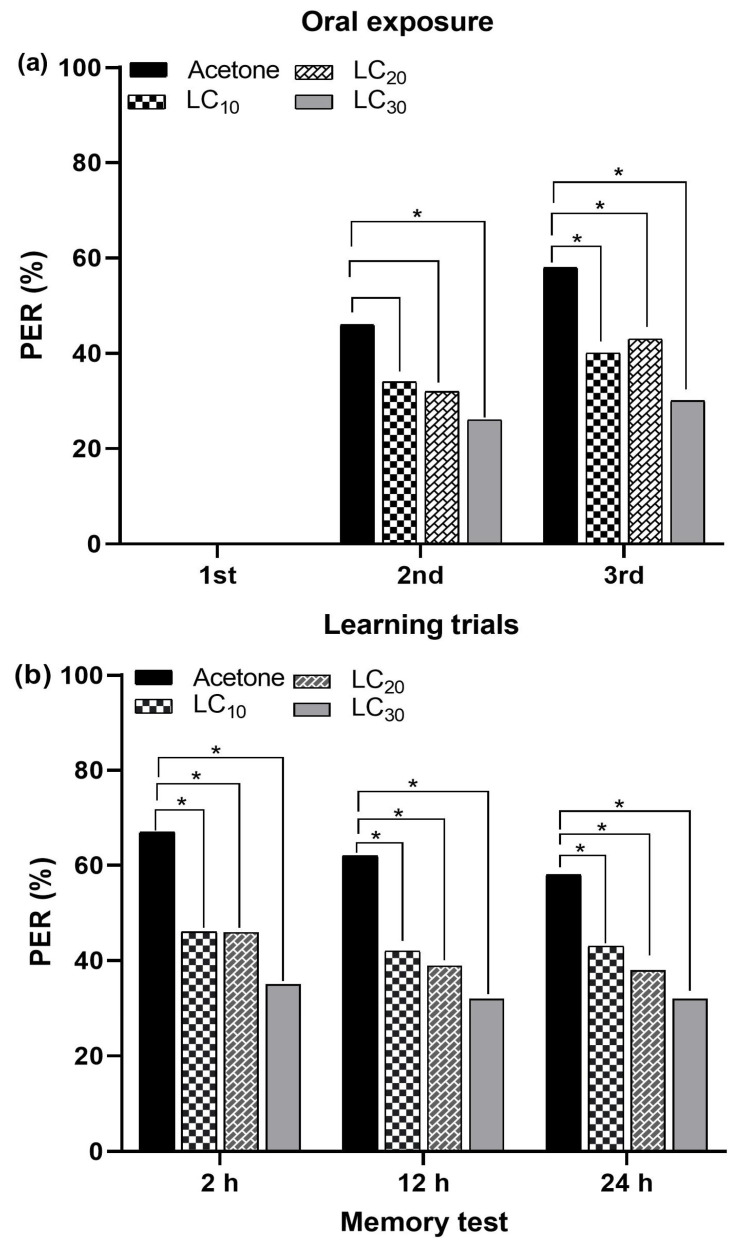
Oral administration of acetamiprid to *Apis mellifera jemenitica*. PER in (**a**) learning trials and (**b**) memory formation. Significant differences between control and insecticide-treated bees are denoted by asterisks (Fisher’s exact test/χ^2^ test; * *p* < 0.05).

The oral administration of sublethal acetamiprid concentrations (LC_10_ = 2.85 ppm, LC_20_ = 4.77 ppm, and LC_30_ = 6.91 ppm) negatively affected the PER during learning and memory formation. No PER was observed during the first learning trial in both the control and treated bees. A significant difference in PER in treated bees was observed during the second and third learning trials for all the tested sublethal acetamiprid concentrations, compared to the control bees (Figure 8a). The memory formation of *A. m. jemenitica* was significantly impaired with oral application of all the tested sublethal concentrations at 2 h, 12 h, and 24 h compared to those of control bees. The control bees exhibited the highest PERs (67%, 62%, and 59%) at 2 h, 12 h, and 24 h, respectively. All tested sublethal concentrations caused an equal reduction in memory formation at 2, 12, and 24 h (Figure 8b). Thus, a lower sublethal concentration (LC_10_) can exert the same effect on memory formation as higher sublethal concentrations (LC_20_ and LC_30_).

## 4. Discussion

Honey bees are under continuous threat due to the extensive use of agrochemicals exposing them to insecticides through various pathways [50,63]. Bees can intake insecticides either orally through contaminated nectar, pollen, and water or topically through direct spray in the agricultural fields [23,83]. Neonicotinoids including acetamiprid are some of the most frequently used pesticides to manage a variety of insect pests [22]. Their potential impact on crucial pollinators has been a subject of concern due to their potential toxic effects [84]. Honey bees can be exposed to insecticides through different routes, such as direct and indirect exposure [85]. Our study highlights that a commercial formulation of acetamiprid (20 SL) exerts drastic effects on honey bee *A. m. jemenitica*, native to Saudi Arabia, which is well known for its heat and drought tolerance [45]. We used two distinct exposure methods (topical and oral) to investigate the mortality and formation of olfactory learning and memory in honey bees.

### 4.1. Mortality of Bees

#### 4.1.1. Topical Exposure

Our data show that topical exposure to acetamiprid at different concentrations (10, 25, 50, and 100 ppm) significantly increased *A. m. jemenitica* mortality at 24 h (10, 10, 24, and 44%, respectively) and 48 h (15, 21, 32, and 46%, respectively). The highest mortalities (44 and 46%) occurred with the field-realistic high concentration (100 ppm) at 24 h and 48 h post-treatment, respectively. The observation that mortality increases with increasing concentrations of acetamiprid is in agreement with previous studies [63,71,86]. The mortality of *A. m. Lamarckii* at 72 h after topical acetamiprid 20 SL application at high concentrations (90 µg/mL) did not exceed 50%, which is in line with our findings [72]. Topically administered acetamiprid (99% purity) at 1 µg/bee resulted in 31.8% mortality after 11 days of chronic exposure, which was not significantly different from the control [87]. The topical application of a commercial acetamiprid formulation (500 ppm) exhibited >20% mortality after 24 h of treatment [88]. In contrast, substantially high *A. mellifera* mortality (92.6–100%) was reported 24 h after topical acetamiprid (200 g/kg) application at a concentration of 10.65–21.42 ng/µL [71]. Mortality reached 83.44% at 24 h after topical application of clothianidin (100 µg/bee), a neonicotinoid, on the thorax [89]. These diverse results can be attributed to the difference in the experimental settings, species and biological status of bees, and potency of the tested insecticides [90]. 

#### 4.1.2. Oral Exposure

Oral exposure of acetamiprid significantly increased the mortality rate of *A. m. jemenitica* at 4, 12, 24, and 48 h post-treatment. Mortality increased proportionally with the concentration of orally administrated acetamiprid, aligned with previous studies [91].

We found the highest mortality (100%) with the field-realistic high concentration (100 ppm) at 24–48 h. A high *A. mellifera* mortality (100%) was also found with the oral ingestion of acetamiprid (200 g/kg) at a concentration of 10.56 ng/μL/bee after 24 h of treatment [71]. Acetamiprid is highly toxic with 95% *A. mellifera* mortality after direct spray on the bees in melon crop (*Cucumis melo* L.) under laboratory conditions [92]. Oral feeding of acetamiprid at a field-realistic concentration (125 µL/100 mL) caused 86.6% mortality at 3 h and 100% mortality at 24–48 h in *A. mellifera* [91]. Our study found comparable mortality (80% at 4 h and 100% at 24–48 h) when utilizing the highly tested field-recommended concentration (100 ppm) of acetamiprid. Likewise, oral feeding of acetamiprid reduced honey bee survival by 84% compared to a control group of bees [93]. Oral exposure to imidacloprid, a neonicotinoid insecticide, at a concentration of 10 ppm also caused high mortality (95% and 100%) in *A. m. jemenitica* at 24 h and 48 h, respectively [94]. 

Contrarily, oral feeding with sublethal doses of commercially available acetamiprid exhibited greater survival and higher thermal tolerance in *A. mellifera* [95]. In addition, oral ingestion of commercial acetamiprid formulation at 100 ppm after starvation caused 50.85% mortality in *A. mellifera* [96]. Badawy et al. [97] reported a lower acetamiprid toxicity towards *A. mellifera*, with mortality below 25% after 24 h at a field-recommended oral dose of 60 mg/L. Oral exposure to acetamiprid at a concentration of 1 µg/bee resulted in 29.3% mortality after 11 days, indicating no significant difference compared to untreated bees [87]. Jacob et al. [98] found that an oral administration of acetamiprid (20 SP) resulted in the least harm to *A. mellifera.* In a feeding test, acetamiprid (8 WP) resulted in mortality below 40% across all tested concentrations (5, 50, and 500 ppm) after 24–48 h of treatment [88]. However, we found *A. m. jemenitica* mortality to be above 50% across the tested concentrations (25, 50, and 100 ppm) during the 24–48 h time period. Numerous studies have reported that other neonicotinoid insecticides (thiacloprid, imidacloprid, and thiamethoxam) can increase mortality and alter bee flight through oral application [35,89,99]. The differences in pesticide formulations (active ingredient or commercial formulation), insecticide potency and concentration, genetic variation in bee species, and honey bee physiological condition may contribute to the variation in honey bee mortality rates [90,98,100].

### 4.2. Food Consumption

*Apis mellifera jemenitica* exposed to food contaminated with acetamiprid exhibited a significant decrease in food consumption compared to the bees fed normal food. The decrease in food consumption was directly associated with the increase in acetamiprid concentration (10, 25, 50, and 100 ppm), and an even a lower quantity of a high concentration was sufficient to induce significant bee mortality. This is in agreement with previous findings wherein the oral feeding of acetamiprid (0.1–0.5 μg/bee) [101] and acetamiprid 20 SL (90 µg/mL) [72] caused significant reductions in sucrose consumption in *A. mellifera*. Thiacloprid (2.0 mg/L), another neonicotinoid, also caused a significant decrease in food intake in *A. mellifera* [102,103]. Contrarily, oral ingestion of acetamiprid-contaminated food did not affect sucrose consumption [93] and caused the highest food intake in bees exposed to acetamiprid (99%) (259.25 mg/L) compared to control bees [104].

### 4.3. Assessing Bee Mortality: Comparing Oral and Topical Exposure Routes

The oral administration of acetamiprid led to a significantly high *A. m. jemenitica* mortality compared to the topical application at all tested time intervals (4, 12, 24, and 48 h). Comparably, Akça and Saruhan [105] quantified that topical exposure to various neonicotinoids had a lesser impact on *A. mellifera* compared to oral exposure after 48 h. Oral exposure to thiamethoxam (neonicotinoid) induced greater mortality in *A. m. intermissa* and *A. m. sahariensis* compared to topical exposure after 24 h of treatment [106]. A similar trend was found in solitary bees (*Osmia bicornis*), where oral exposure to acetamiprid (20 SP) resulted in a higher mortality than after topical exposure [107]. Conversely, the mortality of *A. mellifera* after contact exposure to acetamiprid (20 SL) was greater than oral exposure at 24–48 h after treatment [72]. In addition, the direct spray of commercial acetamiprid (0.06 g a.i./L) onto melon crop, *Cucumis melo* L., resulted in 100% mortality in *A. mellifera*, compared to 47.6% mortality in bees that were orally fed an insecticide-contaminated diet [92].

### 4.4. Lethal Concentrations of Acetamiprid

Determining an insecticide’s lethal concentration (LC) is vital to mitigating the risks to non-target insects and assessing its potential impact on bees [63,108]. In the current study, the LC_50_ values were 160.33 ppm and 141.94 ppm at 24 and 48 h, respectively, after the topical exposure of *A. m. jemenitica* to commercial acetamiprid (20SL). Numerous studies have reported different LC_50_ values, such as 5.26 ng/µL or 5.26 ppm/bee [71], 1.69 μg/bee [97], and 7.1 μg/bee [109] at 24 h in different experimental setups of topical acetamiprid application on *A. mellifera*. One LC_50_ after the chronic topical exposure (48 h to 7 days) of acetamiprid to *A. mellifera* was 2.51 mg/liter (2.57 ppm) [110]. For a single topical application of acetamiprid, the LC_50_ value was 1.17 × 10^5^ ppm after 48 h of treatment [111]. Our data show that the LC_50_ values of acetamiprid were 12.76 ppm and 11.06 ppm after 24 and 48 h of oral exposure, respectively. Previous studies have expressed different LC_50_ values of 4.70 ng/µL or 4.70 ppm [71] and 114.72 ng/bee [111] in *A. mellifera.* Acute oral exposure to acetamiprid (99% purity) in a 50% sucrose solution led to an LD_50_ of 63.1 μg/bee 48 h post-treatment in *A. mellifera* [112]. Thiamethoxam, another neonicotinoid, revealed LC_50_ values of 12.29 ng/bee for *A. m. intermissa* and 13.34 ng/bee for *A. m. sahariensis* after 24 h of oral insecticide ingestion [106]. The discrepancies in lethal thresholds may be due to various factors, including honey bee species, age, seasonal conditions, insecticide formulations, experimental protocols, etc. [63,113].

### 4.5. Honey Bee Associative Learning

Olfactory conditioning in bees was employed to evaluate their cognitive functions after pesticide exposure. Notable impairments in learning and memory formation were displayed in the bees subjected to sublethal concentrations of acetamiprid through topical and oral routes. Consequently, acetamiprid can compromise foraging abilities (learning and memory), thereby negatively impacting colony health.

#### 4.5.1. Acetamiprid Topical Exposure: Learning and Memory Formation

Sublethal concentrations of acetamiprid, 15.23 ppm (LC_10_), 34.18 (LC_20_), and 61.20 ppm (LC_30_), caused reductions in the PER of *A. m. jemenitica* during the learning trials and subsequent memory formation after topical exposure. The insecticide-treated bees exhibited decreased PERs in both the learning trials (2nd–3rd) and memory formation (2–24 h) compared to the untreated bees. Comparably, the 0.5, 1, and 2 μg/bee applications of acetamiprid to the thorax decreased the PER of *A. mellifera*, negatively impacting foragers’ ability to return to their hives and modifying the expression levels of genes involved in learning and memory [5]. Likewise, sublethal doses (0.1 and 1 ng/bee) of thiamethoxam, a neonicotinoid, induced decreases in learning and memory in *A. mellifera* through contact exposure [87]. 

#### 4.5.2. Acetamiprid Oral Exposure: Learning and Memory Formation

The sublethal concentrations of acetamiprid (2.85, 4.77, and 6.91 ppm: LC_10_, LC_20_, and LC_30_, respectively) administered through oral exposure significantly reduced the PERs of *A. mellifera jemenitica* during the learning trials and subsequent memory formation. The treated bees showed decreased learning and memory formation (2–24 h) in comparison to the untreated bees. Notably, the reduction impact of the LC_30_ concentration on PER was comparatively greater than that of LC_10_ and LC_20_. Consistently, oral exposure to acetamiprid (1 μg/bee) negatively affected the long-term memory (24–48 h) of *A. mellifera* [101]. Feeding bees with acetamiprid at 100 ng/bee 24 h before learning impaired memory (24 h), while 10 ng/bee had no effect. However, bees fed the insecticide during learning showed impaired 24 h memory at both concentrations [114]. Interestingly, our study showed an effect on memory when sublethal concentrations of acetamiprid were fed 30 min prior to the learning trials. The oral feeding of acetamiprid (1 and 5 ppm) can also cause significant disorientation accompanied with reduced bee returns [115]. Oral ingestion of imidacloprid at 0.1 ng/bee impaired the olfactory learning and long-term memory (1–17 h) in Asian honey bees, *Apis cerana* [31]. It also negatively affected the bee foraging and pollination, and reduced colony fitness [30,31].

Similarly, thiacloprid and clothianidin (neonicotinoids) also impaired memory function in *A. m. carnica* at 24 h [116,117]. In addition, prolonged exposure to thiacloprid leads to neuronal apoptosis and the downregulation of memory-related genes, resulting in impaired learning and memory in *A. mellifera* [103]. Long-term exposure to acetamiprid may lead to an accumulation of insecticides in honey bees over time, eventually exacerbating the honey bees’ potential and tasks [118]. The effects of agrochemicals on honey bees may differ based on the physiological status of the bees, including nutritional conditions, bee age, effects on nursing bees and seasons, and disease infection [119]. Insecticidal exposure in bees may also be affected by factors such as detoxification, sensitivity, and resistance [120]. The detoxification of neonicotinoids in bee bodies involves metabolic enzymes in the bee stomach, gene expression, and dietary factors [121,122]. These factors may contribute to the differential effects of agrochemicals on honey bees. Collectively, the implementation of monitoring, training, and educational programs is imperative for the proper utilization of these chemicals, with the goal of mitigating the adverse effects of pesticides on non-target pollinators, including honey bees.

## 5. Conclusions

The research findings demonstrate that acetamiprid, a known neonicotinoid insecticide, exhibits deleterious effects on the survival and olfactory cognitive functions of the Saudi-native honey bee, *A. m. jemenitica*. Acetamiprid led to an increase in the bees’ mortality and a reduction in proboscis extension response during learning and memory formation through both topical and oral exposure routes. The field-realistic (100 ppm) and serially diluted concentrations of acetamiprid (50, 25, and 10 ppm) resulted in significant mortality at 4, 12, 24, and 48 h post-treatment, with the peak bee mortality observed at 24 and 48 h following both exposure routes. Bee mortality increased with higher insecticide concentrations across all the tested time intervals, showing a concentration-dependent pattern. Sublethal concentrations (LC_10_, LC_20_ and LC_30_) of acetamiprid negatively affected the learning and memory of honey bees. The findings highlight the acute risks associated with acetamiprid use, including high mortality rates, reduced food intake, and impaired learning and memory formation in bees. These results emphasize the importance of implementing careful management practices to mitigate insecticide-related harm to honey bee populations. Moreover, continuous monitoring efforts across Saudi Arabia’s agricultural regions are imperative to assess and address the potential adverse effects of insecticides on honey bees.

## Figures and Tables

**Table 1:** Lethal concentrations (LC_50_ and LC_90_) following topical and oral exposure to acetamiprid in *Apis mellifera jemenitica*.

Exposure Route	Time (h)	LC_50_ (ppm)	LC_90_ (ppm)
Topical	12	172.67	1154.89
	24	160.33	1687.27
	48	141.94	3052.37
Oral	12	16.70	77.02
	24	12.76	57.06
	48	11.06	52.51

**Table 2:** Sublethal acetamiprid concentrations 12, 24, and 48 h after treatment.

Time (h)	Sublethal Concentration (ppm)					
	Topical Exposure			Oral Exposure		
	LC_10_	LC_20_	LC_30_	LC_10_	LC_20_	LC_30_
12	25.81	49.57	79.34	3.62	6.12	8.93
24	15.23	34.18	61.20	2.85	4.77	6.91
48	6.60	18.92	40.44	2.33	3.97	5.85

## Data Availability

Data are provided within the article.
